# Genomic Insights into the Role of cAMP in Carotenoid Biosynthesis: Enhancing β-Carotene Production in *Escherichia coli* via *cyaA* Deletion

**DOI:** 10.3390/ijms252312796

**Published:** 2024-11-28

**Authors:** Soon-Jae Kwon, Chan Bae Park, Pyung Cheon Lee

**Affiliations:** 1 Department of Molecular Science and Technology, Advanced College of Bio-Convergence Engineering, Ajou University, Woncheon-dong, Yeongtong-gu, Suwon 16499, Republic of Korea; soon87jae@ajou.ac.kr; 2 Department of Physiology, Ajou University School of Medicine, Suwon 16499, Republic of Korea

**Keywords:** β-carotene, cAMP, *cyaA*, mutation

## Abstract

The gamma-ray-induced random mutagenesis of an engineered β-carotene-producing *Escherichia coli* XL1-Blue resulted in the variant Ajou 45, which exhibits significantly enhanced β-carotene production. The whole-genome sequencing of Ajou 45 identified 55 mutations, notably including a reduction in the copy number of *cyaA*, encoding adenylate cyclase, a key enzyme regulating intracellular cyclic AMP (cAMP) levels. While the parental XL1-Blue strain harbors two copies of *cyaA*, Ajou 45 retains only one, potentially leading to reduced intracellular cAMP concentrations. This reduction may alleviate catabolite repression and redirect metabolic flux toward the β-carotene biosynthesis pathway. To validate this mechanistic insight, a targeted *cyaA* knockout was engineered in XL1-Blue, and its β-carotene production and growth phenotypes were compared with those of Ajou 45 and XL1-Blue. The findings demonstrated that a reduced *cyaA* copy number substantially enhances β-carotene biosynthesis by modulating cAMP-mediated regulatory networks. This study highlights the efficacy of integrating random mutagenesis with integrative genomic analysis for microbial strain engineering and presents a novel strategy for enhancing carotenoid production in *E. coli*.

## 1. Introduction

**Figure 1:**
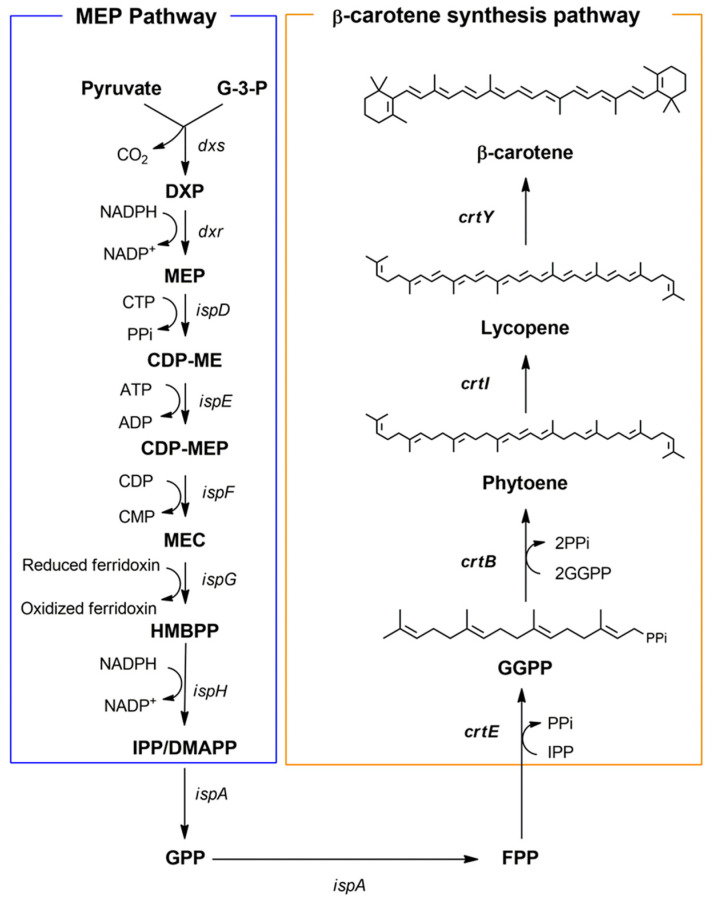
Schematic representation of the endogenous MEP pathway and the heterologous β-carotene biosynthesis pathway in recombinant *Escherichia coli* expressing β-carotene biosynthetic enzymes. The left panel illustrates the MEP (methylerythritol phosphate) pathway, which synthesizes isoprenoid precursors starting from pyruvate and glyceraldehyde 3-phosphate (G-3-P). Key intermediates in this pathway include DXP (1-deoxy-D-xylulose-5-phosphate), MEP (2-C-methyl-D-erythritol-4-phosphate), CDP-ME (4-diphosphocytidyl-2-C-methyl-D-erythritol), CDP-MEP (4-diphosphocytidyl-2-C-methyl-D-erythritol 2-phosphate), MEC (2-C-methyl-D-erythritol-2,4-cyclodiphosphate), and HMBPP (1-hydroxy-2-methyl-2-butenyl 4-diphosphate). This pathway culminates in the production of isopentenyl pyrophosphate (IPP) and dimethylallyl pyrophosphate (DMAPP), which are interconverted by idi. These intermediates are further converted into geranyl pyrophosphate (GPP) and farnesyl pyrophosphate (FPP) by ispA. The right panel depicts the β-carotene biosynthesis pathway. FPP is converted into geranylgeranyl pyrophosphate (GGPP) by crtE, which is then converted into phytoene by CrtB. Phytoene is desaturated into lycopene by CrtI, and finally cyclized into β-carotene by CrtY. Arrows represent enzymatic steps, with corresponding genes labeled.

Carotenoids, including β-carotene, are valuable pigments widely used in industries such as food, cosmetics, and pharmaceuticals due to their antioxidant properties and role as precursors for vitamin A [1,2,3]. β-carotene is naturally produced by plants (e.g., carrots and sweet potatoes), algae (e.g., *Dunaliella salina*), and certain microorganisms such as fungi and photosynthetic bacteria [1]. The microbial production of β-carotene has attracted significant attention as a sustainable and scalable alternative to chemical synthesis, offering advantages in terms of environmental sustainability and bioavailability [4,5]. In heterologous microbial hosts, β-carotene is synthesized from precursors generated via the endogenous isoprenoid pathway, specifically the methylerythritol phosphate (MEP) pathway in *E. coli* (Figure 1). In this pathway, the isoprenoid precursors isopentenyl pyrophosphate (IPP) and dimethylallyl pyrophosphate (DMAPP) are converted into geranylgeranyl pyrophosphate (GGPP), which is subsequently transformed into β-carotene through a series of enzymatic steps introduced via heterologous carotenogenic genes [6]. Despite advances in metabolic engineering and synthetic biology to optimize carotenoid biosynthesis, β-carotene production in heterologous hosts, including *E. coli,* is often constrained by global metabolic regulators. Among these, the cyclic AMP (cAMP) receptor protein (CRP) complex plays a critical role in regulating metabolic flux and gene expression, posing a significant challenge for enhancing carotenoid biosynthesis.

Adenylate cyclase, encoded by the *cyaA* gene, catalyzes the synthesis of cAMP, a key signaling molecule that interacts with CRP to regulate the expression of genes involved in carbon metabolism, biosynthesis, and other cellular processes [7]. The cAMP-CRP complex is known to repress secondary metabolite production, including carotenoids, by modulating primary metabolic pathways and limiting the availability of precursors for secondary metabolism [8,9]. This regulatory mechanism suggests that reducing intracellular cAMP levels—such as through the targeted deletion of *cyaA*—may alleviate catabolite repression, thereby redirecting metabolic flux toward β-carotene biosynthesis.

Although targeted genetic modifications are widely employed to enhance carotenoid yields, random mutagenesis remains a valuable complementary approach, enable the generation of genetic diversity without requiring prior knowledge of specific target genes. This approach facilitates the identification of novel beneficial mutations that enhance metabolic performance [10,11]. By integrating random mutagenesis with whole-genome sequencing, it is possible to pinpoint mutations underlying phenotypic improvements and validate their contributions through targeted genetic engineering.

In this study, we employed gamma-ray-induced random mutagenesis to enhance β-carotene production in a recombinant *E. coli* XL1-Blue strain harboring the plasmid *pUC19-crtEBIY*. This approach led to the isolation of a mutant strain, Ajou 45, which exhibited significantly enhanced β-carotene production compared to the parental strain. The whole-genome sequencing of Ajou 45 revealed multiple mutations, including a notable reduction in the copy number of the *cyaA* gene, from two copies in XL1-Blue to one in Ajou 45. Given the established role of cAMP in global metabolic regulation, we hypothesized that this reduction contributed to the enhanced β-carotene production observed in Ajou 45. To test this hypothesis, we constructed a *cyaA* knockout strain from the parental XL1-Blue, and compared its β-carotene production and growth phenotypes to those of Ajou 45 and XL1-Blue.

Our findings provide new insights into the role of cAMP regulatory networks in carotenoid biosynthesis and highlight the utility of integrating random mutagenesis with genomic analysis for strain improvement.

## 2. Results

### 2.1. Enhanced β-Carotene Production and Phenotypic Traits of Mutant Ajou 45

**Figure 2:**
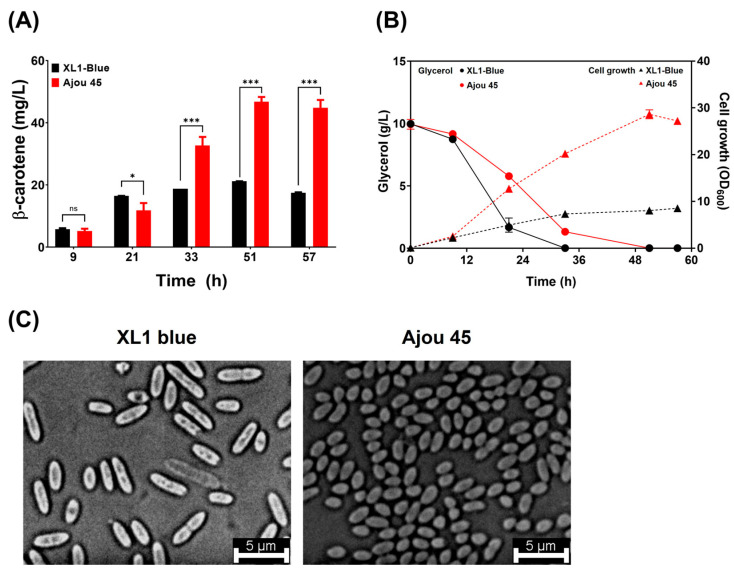
Comparison of β-carotene production, cell growth, glycerol consumption, and cell morphology between XL1-Blue and Ajou 45 cultured in TB medium supplemented with 1% (*w/v*) glycerol. (**A**) Time-course β-carotene production in XL1-Blue and Ajou 45 strains. Statistical significance was assessed across three independent experiments. Error bars denote standard deviation (SD); ns, no significance, * *p* < 0.05, *** *p* < 0.001. (**B**) Cell growth (OD_600_) and glycerol consumption profiles of XL1-Blue and Ajou 45 strains during cultivation. (**C**) Microscopic images of cell morphology for XL1-Blue and Ajou 45 strains, with scale bars representing 5 μm.

The mutant strain Ajou 45 was systematically characterized for its β-carotene production, growth kinetics, glycerol consumption rate, and phenotypic difference relative to the parental XL1-Blue strain. Both strains were aerobically cultured in TB medium supplemented with 10 g/L glycerol as a carbon source. The time-course quantification of β-carotene production revealed that Ajou 45 consistently exhibited significantly higher production levels than XL1-Blue after 33 h of cultivation (Figure 2A). Ajou 45 achieved the highest β-carotene production of 46.8 ± 1.9 mg/L at 51 h, exceeding more than twice the production observed in XL1-Blue (21.2 ± 0.9 mg/L), demonstrating its enhanced carotenoid biosynthetic potential.

The growth analysis revealed that Ajou 45 achieved a higher maximum optical density at 600 nm (OD_600_) than XL1-Blue. Ajou 45 reached an OD_600_ of 28.6 at 51 h, reflecting a 3.4-fold increase compared to the OD_600_ of 8.5 observed in XL1-Blue (Figure 2B). However, Ajou 45 exhibited a slower glycerol consumption rate, requiring 51 h for complete glycerol consumption, whereas XL1-Blue depleted glycerol within 33 h. Despite the slower glycerol consumption rate, Ajou 45 sustained superior β-carotene production levels. Microscopic analysis revealed a notable phenotypic difference between the two strains. Ajou 45 cells were smaller in size compared to XL1-Blue cells (Figure 2C). This phenotypic change, along with observed differences in metabolic and growth behaviors, suggest that the genetic mutations in Ajou 45 may have contributed to its enhanced β-carotene production and altered cellular characteristics.

### 2.2. Whole-Genome Analysis of Parental XL1-Blue and Mutant Ajou 45

To elucidate the genetic basis underlying the observed differences in β-carotene production, cell growth, glycerol consumption, and cell size between parental XL1-Blue and its mutant strain Ajou 45, a comprehensive genomic analysis was performed with high-resolved genomes.

First, the sequence of the *pUC19-crtEBIY* plasmid isolated from Ajou 45 was analyzed to determine whether mutations in the carotenogenic genes, expression regulatory elements, or plasmid copy number contributed to the phenotypic changes. Sequencing confirmed that the plasmid sequence in Ajou 45 was identical to that of the parental XL1-Blue strain. This finding indicates that the observed phenotypic differences in Ajou 45 were solely attributable to chromosomal mutations. 

Next, the genomes of XL1-Blue and Ajou 45 were sequenced using both PacBio and Illumina sequencing technology to identify single nucleotide polymorphisms (SNPs) and structural variations. The genome of XL1-Blue was determined to consist of a circular chromosome of 4,633,492 base pairs (bp) with a G + C content of 50.8%. In contrast, the genome of Ajou 45 exhibited a slightly smaller circular chromosome of 4,632,389 bp, while maintaining the same G+C content of 50.8% (Table 1). Annotation performed using the RAST server revealed 4443 coding DNA sequences (CDSs) in XL1-Blue, compared to 4425 CDSs in Ajou 45, indicating a slight reduction in the coding capacity of the mutant strain.

The phylogenetic analysis of XL1-Blue and Ajou 45, in comparison with seven reference *Escherichia coli* genomes, revealed that both strains clustered within the same clade, further confirming that Ajou 45 was derived from parental XL1-Blue (Supplementary Figure S1). These findings highlight the chromosomal basis of the phenotypic changes observed in Ajou 45, which are associated with its improved metabolic efficiency and β-carotene production.

The subsystem categorization of the CDSs revealed significant differences in functional groups between Ajou 45 and its parental XL1-Blue strain, as illustrated in Supplementary Figure S2. Genomic analysis identified a total 55 mutations in Ajou 45 (Supplementary Table S1), including 11 in well-characterized protein-coding genes with potential functional significance (Table 2). Among these, a prominent 2655 bp deletion was observed in *cyaA*, which encodes adenylate cyclase, reducing its copy number from two in XL1-Blue to one in Ajou 45. This deletion is predicted to decrease intracellular cAMP levels, potentially leading to the partial derepression of key metabolic pathways and enhancing metabolic flexibility [7]. Additionally, a mutation in *manB*, a gene involved in cell wall synthesis, may explain the smaller cell size observed in Ajou 45, as alterations in *manB* can affect cell wall integrity and morphology [12]. Interestingly, mutations in *argF, ilvD*, and *mtn* may impact amino acid biosynthesis and nitrogen metabolism in Ajou 45, potentially contributing to its metabolic reprogramming. Collectively, these mutations provide insights into the genetic basis for the phenotypic alterations and the enhanced heterologous β-carotene production observed in Ajou 45. Systematic studies of these mutated genes (Table 2) can further elucidate the mechanisms underlying these functional and phenotypic changes.

### 2.3. Effect of cyaA Gene Deletion in XL1-Blue on β-Carotene Production

**Figure 3:**
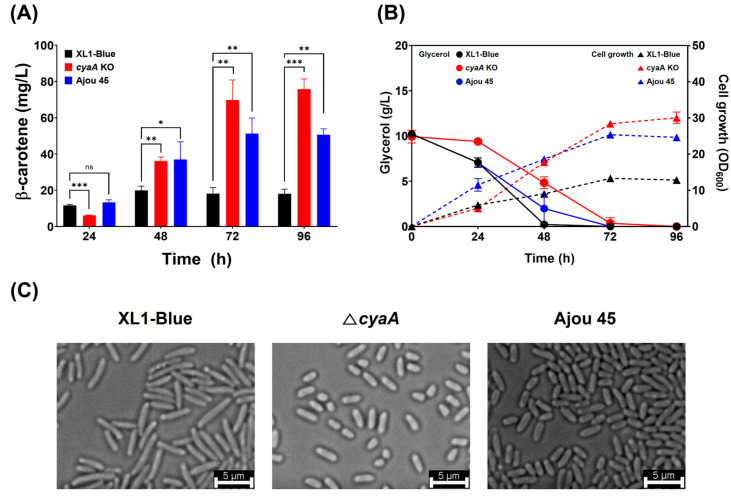
Comparison of β-carotene production, cell growth, glycerol consumption, and cell morphology between XL1-Blue, *cyaA* knockout (KO), and Ajou 45 strains. (**A**) Time-course β-carotene production in XL1-Blue, *cyaA* KO, and Ajou 45 strains. Statistical significance was assessed across three independent experiments. Error bars denote standard deviation (SD); ns, no significance, * *p* < 0.05, ** *p* < 0.01, *** *p* < 0.001. (**B**) Cell growth (OD_600_) and glycerol consumption profiles of XL1-Blue, *cyaA* KO, and Ajou 45 strains during cultivation. (**C**) Microscopic images of cell morphology for the three strains, with scale bars representing 5 μm.

Among the 55 mutations identified in the genome of Ajou 45, the deletion of the *cyaA* gene was highlighted as particularly significant due to the critical role of adenylate cyclase, encoded by *cyaA*, in regulating metabolic pathways, including carotenoid biosynthesis [8,9]. To investigate the impact of *cyaA* deletion on β-carotene production, a *cyaA* knockout (KO) strain was constructed from the XL1-Blue (Supplementary Figure S3). The resulting *cyaA* KO strain (one genomic copy of *cyaA*), along with the wild-type XL1-Blue (two copies of *cyaA*) and the mutant Ajou 45 (one copy of *cyaA*), was cultured in TB medium supplemented with 1% (*w/v*) glycerol to assess β-carotene production and associated phenotypic traits (Figure 3).

The quantitative analysis of β-carotene production (Figure 3A) revealed significant differences among the strains. After 96 h of cultivation, the XL1-Blue strain produced 19.9 ± 2.4 mg/L of β-carotene, while the *cyaA KO* strain exhibited a substantially higher production level of 75.8 ± 5.6 mg/L, representing an approximately 3.8-fold increase. Ajou 45 demonstrated an intermediate production level of 51.3 ± 8.6 mg/L. These results indicate that *cyaA* exerts a repressive role in β-carotene biosynthesis, and its deletion significantly enhanced β-carotene production. However, the lower production observed in Ajou 45 compared to the *cyaA* KO strain suggests that additional mutations in the Ajou 45 genome may partially mitigate the positive effect of the *cyaA* deletion on β-carotene biosynthesis. 

Cell growth (Figure 3B) followed a similar trend to β-carotene production levels. The XL1-Blue reached a maximum OD_600_ of 13.3, while the *cyaA* KO strain demonstrated significantly enhanced growth, achieving a maximum OD_600_ of 30.0. Ajou 45 exhibited intermediate growth, with a maximum of OD_600_ of 25.3. These findings suggest that the deletion of *cyaA* not only enhances β-carotene production but also promotes greater cell growth, likely due to the depression of the metabolic pathway regulated by cAMP signaling. Distinct glycerol consumption profiles were observed among the strains. XL1-Blue consumed glycerol more rapidly than both *cyaA* KO and Ajou 45, while the *cyaA* KO strain exhibited the slowest glycerol consumption despite its higher growth and β-carotene biosynthesis. This slower carbon utilization in *cyaA KO* correlates with increased β-carotene biosynthesis, suggesting a reallocation of metabolic fluxes toward carotenoid biosynthesis. 

Microscopic analysis (Figure 3C) provided further insights into the physiological effect of *cyaA* deletion. The *cyaA* KO strain exhibited smaller cell size compared to XL1-Blue, resembling the cell morphology of Ajou 45. These findings suggest that alterations in cAMP-mediated signaling pathways, caused by *cyaA* deletion, may influence cell division and expansion. Additionally, the observed phenotypic and metabolic changes provide a clearer understanding of the role of *cyaA* deletion in enhancing β-carotene biosynthesis and altering cellular physiology in Ajou 45.

### 2.4. Effect of cAMP Supplementation on β-Carotene Production and Metabolic Traits

**Figure 4:**
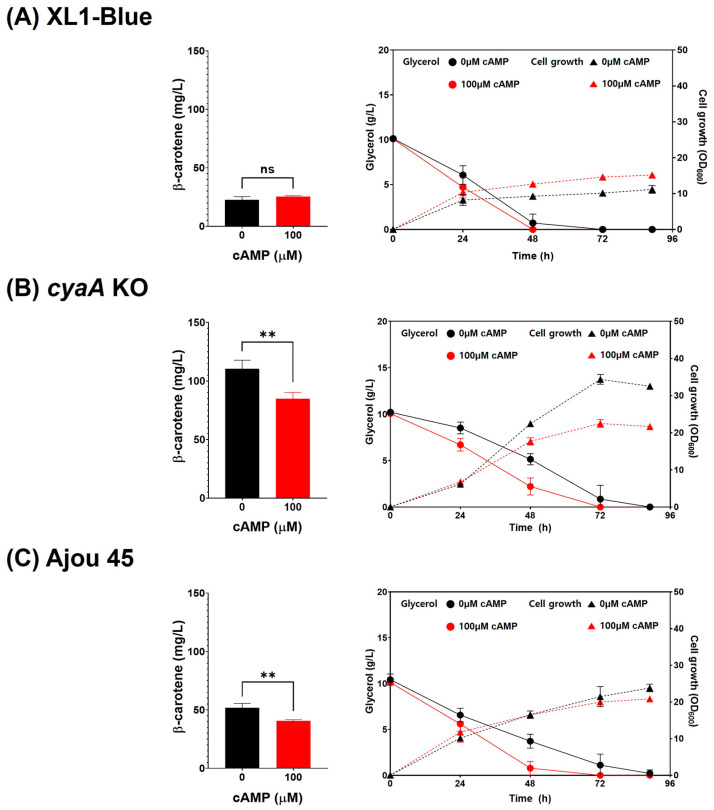
Effect of cAMP supplementation on β-carotene production, cell growth, and glycerol consumption in XL1-Blue, *cyaA* KO, and Ajou 45 strains. (**A**) β-carotene production (**left**) and cell growth/glycerol consumption (**right**) in XL1-Blue with 0 µM and 100 µM cAMP supplementation. (**B**) β-carotene production (**left**) and cell growth/glycerol consumption (**right**) in *cyaA* KO strain with 0 µM and 100 µM cAMP supplementation. (**C**) β-carotene production (**left**) and cell growth/glycerol consumption (**right**) in Ajou 45 with 0 µM and 100 µM cAMP supplementation. Error bars represent standard deviation (SD) from three independent experiments. Statistical significance: ns = not significant, ** *p* < 0.01.

The role of cAMP in regulating β-carotene production was further examined by cultivating wild-type XL1-Blue, *cyaA* KO, and Ajou 45 strains in TB medium supplemented with 100 µM cAMP. In the XL1-Blue strain, the addition of 100 µM cAMP had no significant effect on β-carotene production (22.7 ± 2.7 mg/L vs. 25.5 ± 0.7 mg/L), suggesting that cAMP does not directly regulate carotenoid biosynthesis in this strain (Figure 4A, left). However, in both the *cyaA* KO and Ajou 45 strains, β-carotene production decreased significantly with cAMP supplementation (from 110.4 ± 7.5 mg/L to 84.7 ± 5.5 mg/L in *cyaA* KO; from 51.7 ± 3.8 mg/L to 40.7 ± 1.1 mg/L in Ajou 45) (Figure 4B,C, left). These findings indicate that elevated cellular cAMP levels suppress β-carotene biosynthesis in both the cyaA KO and Ajou 45 strains with reduced *cyaA* function, both of which possess only one genomic copy of *cyaA*.

The impact of cAMP supplementation on cell growth revealed distinct strain-specific responses. In the XL1-Blue strain, cAMP supplementation led to an increase in maximum OD_600_ (from 11.2 ± 1.1 to 15.2 ± 0.4), indicating a cAMP-dependent enhancement of cell growth (Figure 4A, right). In contrast, both the *cyaA* KO and Ajou 45 strains exhibited reduced cell growth upon cAMP supplementation, particularly in the *cyaA* KO strain, where OD_600_ decreased from 34.4 ± 1.3 to 22.5 ± 1.1 at 72 h of culture (Figure 4B,C, right). This repressive effect on cell growth in two strains with reduced *cyaA* function aligns with earlier findings (Figure 3B) that *cyaA* deletion enhances cell growth by derepressing cAMP-regulated pathways.

Glycerol consumption profiles further supported the regulatory role of cAMP. Across all strains, cAMP supplementation accelerated glycerol consumption rates, with the effect being most pronounced in the *cyaA* KO and Ajou 45 strains (Figure 4A–C, right). While an elevated cAMP level promoted glycerol uptake, it simultaneously suppressed β-carotene biosynthesis, particularly in the two strains with reduced functional *cyaA*.

Overall, these findings confirm that cAMP acts as a repressor of β-carotene biosynthesis in heterologous *E. coli* hosts, particularly in strains with reduced *cyaA* function. The increased β-carotene production observed in the absence of cAMP underscore the potential of reducing intracellular cAMP levels as a metabolic strategy to enhance carotenoid biosynthesis. Thus, *cyaA* and its role in cAMP synthesis play a pivotal role in modulating both β-carotene production and cellular metabolism, as demonstrated in the *cyaA* KO and Ajou 45 strains.

## 3. Discussion

This study demonstrated that the gamma-ray-induced random mutagenesis of *E. coli* XL1-Blue successfully generated the mutant strain Ajou 45, which exhibited significantly enhanced β-carotene production. Comparative genomic analysis between XL1-Blue and Ajou 45 identified 55 mutations in the genome of Ajou 45, including 11 mutations in well-characterized protein-coding genes. Among these, the one-copy deletion of *cyaA*, encoding adenylate cyclase, emerged as a key mutation responsible for the observed phenotypic changes. Adenylate cyclase plays a central role in cyclic AMP (cAMP) synthesis, and the loss of *cyaA* in both Ajou 45 and *cyaA* KO resulted in a substantial increase in β-carotene production. These findings provide strong evidence supporting the hypothesis that cAMP acts as a repressor of heterologous β-carotene biosynthesis. This result is consistent with previous studies showing that the cAMP-CRP complex plays a critical regulatory role in *E. coli* metabolic pathways, particularly those associated with secondary metabolite biosynthesis such as carotenoids [13].

The regulatory role of cAMP in bacterial metabolism is well established, particularly through its interaction with CRP, which regulates global metabolic processes, including glycolysis, the TCA cycle, and carbon catabolism [14]. Reduced intracellular cAMP levels have been shown to derepress biosynthetic pathways, redirecting metabolic fluxes toward secondary metabolites like carotenoids by alleviating repression on key genes involved in isoprenoid precursor synthesis [15,16]. In Ajou 45, the decreased cAMP levels likely facilitated metabolic reallocation, enhancing flux through the methylerythritol phosphate (MEP) pathway, which is crucial for precursor availability in β-carotene biosynthesis.

Distinct phenotypic changes observed in Ajou 45 and *cyaA* KO further underscore the role of cAMP in balancing growth and metabolite production. Ajou 45 and *cyaA* KO exhibited slower glycerol consumption and altered growth dynamics compared to the parental strain XL1-Blue. This metabolic shift aligns with the notion that reduced cAMP levels prioritize carotenoid biosynthesis over rapid carbon catabolism [17]. Slower growth and delayed glycerol consumption in Ajou 45 and *cyaA* KO suggest improved carbon flux distribution toward β-carotene synthesis rather than rapid cell proliferation. Such phenotypic adaptations reflect the broader regulatory impact of *cyaA* deletion on cellular metabolism, offering insights into how global metabolic regulators influence secondary metabolite production. Although both Ajou 45 and the targeted *cyaA* knockout strains retain an additional copy of the *cyaA* gene, the observed phenotypic changes suggest that this copy may be transcriptionally inactive or downregulated. The quantification of *cyaA* expression levels from this additional copy will be pursued in future studies to provide further mechanistic insights into the regulation of cAMP levels and β-carotene biosynthesis.

Despite the significant findings, the genetic complexity of Ajou 45 suggests that multiple genomic changes contribute to its enhanced β-carotene production and altered phenotypic characteristics. To gain a deeper understanding of the regulatory mechanisms underlying these changes, system-level analyses such as RNA sequencing (RNA-Seq)-based transcriptomics are necessary. Transcriptomic studies would provide insights into how *cyaA* deletion and other mutations collectively influence gene expression, regulatory networks, and metabolic reallocation. Integrating transcriptomic data with genomic analysis would facilitate a systems-level understanding of the metabolic shifts driving enhanced β-carotene production and could reveal synergistic regulatory targets for further optimization.

Beyond advancing our understanding of cAMP’s role in carotenoid biosynthesis, this study establishes a framework for optimizing microbial hosts for secondary metabolite production. Modulating cAMP levels or engineering the CRP-cAMP signaling pathway represents a promising strategy for improving carotenoid production and potentially other secondary metabolites. Future studies should explore potential synergies between cAMP-CRP regulation and other global regulators, such as RpoS or FNR, to maximize metabolic efficiency and product yields [16,17].

In conclusion, the deletion of *cyaA* in Ajou 45 significantly enhanced β-carotene production, confirming the repressive role of cAMP in carotenoid biosynthesis. This study highlights the potential of integrating random mutagenesis, genomic analysis, and transcriptomic insights to improve microbial strains for industrial biotechnology applications. These findings provide a robust framework for optimizing microbial hosts to produce secondary metabolites, with implications for scalable and efficient biotechnological processes.

## 4. Materials and Methods

### 4.1. Strains and Media

The *Escherichia coli* strains used in this study are listed in Table 3*. E. coli* XL1-Blue was cultured at 30 °C in Luria Broth (LB) medium, which contained 10 g/L tryptone, 5 g/L yeast extract, and 5 g/L NaCl. Additionally, TB (Terrific Broth) medium was used, consisting of 12 g/L tryptone, 24 g/L yeast extract, 9.4 g/L K_2_HPO_4_, 2.2 g/L KH_2_PO_4_, and 10 g/L glycerol (sterilized separately). When required, the appropriate antibiotics—ampicillin (100 mg/L) or kanamycin (30 mg/L)—were added. The crtEBIY genes were amplified from the genomic DNA of *Pantoea agglomerans* ACTC 2479 and cloned into the pUC19 vector to construct the pUC19-crtEXYIBZ plasmid.

### 4.2. Mutagenesis of β-Carotene-Producing E. coli 

Recombinant *E. coli* XL1-Blue cells harboring plasmid pUC19-crtEBIY, which contains a suite of heterologous carotenogenic genes (*crtE, crtB, crtI*, and *crtY*) necessary for the biosynthesis of β-carotene, were subjected to mutagenesis using a ^60^Co gamma-ray source. Initial screening for β-carotene overproducing mutants was conducted on agar plates by selecting colonies that exhibited intensified yellow pigmentation compared to the baseline color of the parent strain. A secondary screening involved quantifying the intensity of yellow coloration in harvested cells to identify strains with elevated β-carotene levels. The mutant strain demonstrating the highest pigment intensity was designated “Ajou 45” and was deposited in the Korean Collection for Type Cultures (KCTC) under accession number KCTC 12683BP.

### 4.3. Culture Conditions in Flask

To compare β-carotene production in flask cultures, *E. coli* strains were pre-cultured in 4 mL of LB medium at 30 °C for 15 h in 15 × 150 mm test tubes. The cultures were then transferred into 20 mL of TB medium in 100 mL flasks and grown for 12 h. Subsequently, 10 mL of the cultured medium was transferred into 100 mL of fresh TB medium containing 0.5 g of CaCO_3_ in 500 mL flasks, with an initial OD_600_ of 0.5. The cells were cultivated at 30 °C with shaking at 250 rpm. For experiments involving cAMP supplementation, 0 or 100 µM of cAMP was added to the TB medium. The concentration of cAMP used for complementation was selected based on previously reported effective ranges for modulating cAMP-CRP activity in *E. coli*. This concentration was chosen to ensure physiological relevance while minimizing potential toxicity or non-specific effects. The initial OD_600_ for these cultures was also set to 0.5, and the cells were incubated under the same conditions.

### 4.4. Carotenoids Extraction and Quantification

Cells were harvested by centrifugation at 4 °C, 4000 rpm, and the resulting pellets were extracted with 400 µL of acetone until all visible pigments were dissolved. The colored supernatants were then extracted with an equal volume of 5N NaCl, followed by pH adjustment to 2 using phosphoric acid. Vortexing was performed after the addition of 800 µL of hexane, followed by centrifugation at 13,000 rpm for 10 min. The hexane layer was collected, and 0.1 g of sodium sulfate was added to remove any residual water. The solvent was evaporated using an EZ2-plus evaporator (Genevac, New York, NY, USA), and the residue was dissolved in 200 µL of acetone. Carotenoid content was quantified by HPLC using an Agilent 1260 HPLC system equipped with a photodiode array detector (DAD) and a Zorbax Eclipse XDB-C18 column (4.6 × 150 mm, 5.0 µm; Agilent Technologies, USA). The mobile phase consisted of acetonitrile/methanol (80:15:5) at a flow rate of 1 mL/min under isocratic conditions. β-carotene was detected at 453 nm, and its concentration was quantified using a standard curve generated with authentic β-carotene (Sigma Aldrich, St. Louis, MO, USA). The results represent the average of three independent experiments.

### 4.5. Microscopic Examination

Samples were analyzed using a Leica DM 2500 light microscope (Leica, Wetzlar, Germany) equipped with phase-contrast optics. Unstained cells were observed at 1000× magnification using an immersion oil objective.

### 4.6. Metabolite Analysis

The concentration of glycerol in the culture supernatants was determined using an Agilent 1200 HPLC system equipped with a refractive index detector (1200 series, Agilent Technologies, Santa Clara, CA, USA) and an Aminex HPX-87H column (Bio-Rad, Hercules, CA, USA). The mobile phase was 4 mM H_2_SO_4_, with a flow rate of 0.7 mL/min and a column temperature of 50 °C.

### 4.7. Construction of cyaA Knockout Strain

The *cyaA* gene knockout in *E. coli* XL1-Blue was generated using the CRISPR-Cas9 system. A single guide RNA (sgRNA) targeting the *cyaA* gene was designed and cloned into the pgRNA plasmid. The *kanamycin resistance* (*kan^R^*) cassette, flanked by 50 bp homology arms corresponding to regions upstream and downstream of *cyaA*, was amplified by PCR and used as the donor template for homology-directed repair (HDR). *E. coli* cells were co-transformed with the pMP11 plasmid (encoding Cas9 and tracrRNA) and the pgRNA_cyaA plasmid. Cells were grown in LB medium containing 100 mg/L ampicillin and 30 mg/L kanamycin at 30 °C. The induction of the CRISPR-Cas9 system was achieved by adding 0.2% arabinose. Homologous recombination between the donor template and the chromosomal *cyaA* locus facilitated the replacement of the *cyaA* gene with the *kan^R^* cassette. Kanamycin-resistant colonies were selected, and the knockout was confirmed by PCR using primers flanking the *cyaA* region (Supplementary Figure S3). For marker removal, the pCP20 plasmid was introduced to express FLP recombinase, and kanamycin-sensitive colonies were selected. The deletion of *cyaA* and the removal of the *kan^R^* cassette were verified by sequencing.

### 4.8. Genome Resequencing and Annotation

Genomic DNA from XL1-Blue and Ajou 45 strains was extracted using a Genomic DNA Kit (Macrogen, Seoul, Republic of Korea). For Illumina sequencing, libraries were prepared following the NEBNext® Ultra™ DNA Library Prep Kit protocol (E7370S, New England Biolabs, UK) and sequenced on the Illumina HiSeq 2000 platform. This generated high-quality paired-end reads with a total of 7,067,287 reads and 713,795,987 bases per pair for XL1-Blue and 6,534,430 reads and 984,082,605 bases per pair for Ajou 45. Long-read sequencing was performed on the Pacific Biosciences (PacBio) RS II single-molecule real-time system, producing approximately 1.1 Gb of raw subreads for XL1-Blue and 1.9 Gb for Ajou 45. The sequencing depth achieved was ~239 × for XL1-Blue and ~413 × for Ajou45 with PacBio, while Illumina sequencing provided an additional depth of ~278× for XL1-Blue (1.28 Gb) and ~345× for Ajou45 (1.59 Gb).

PacBio subreads were assembled de novo using a Canu v1.3 assembler [19], with a genome size parameter of 4.6 Mb. Circular genome assembly was achieved by identifying and trimming overlapping contig ends using Circlator v1.5.5 [20]. The error correction of the PacBio assemblies was performed iteratively using Pilon v1.24 [17], which utilized Illumina paired-end reads preprocessed with Sickle v1.33 (https://github.com/najoshi/sickle, assessed on 14 March 2022). Genome annotation and functional predictions were carried out using the RAST server (https://rast.nmpdr.org/, assessed on 8 November 2022).

For comparative genomic analysis, reference genomes of *Escherichia coli* strains were retrieved from the NCBI Genome Database, including XL1-Blue (CP081007.1), K-12 MG1655 (NC_000913), BL21-Gold (DE3) pLysS AG (NC_012947.1), K-12 W3110 (CP017979.1), BW25113 (CP069134.1), K-12 (CP047127.1), and DH5α (CP080399.1). Phylogenetic relationships were inferred using the neighbor-joining method in MEGA 11.0 software based on whole-genome alignments.

To assess the integrity of the pUC19-crtEXYIBZ plasmid, plasmid DNA was isolated from the Ajou 45 strain using a plasmid purification kit (GeneAll, Seoul, Republic of Korea) and sequenced using Sanger sequencing (Macrogen, Seoul, Republic of Korea).

## Figures and Tables

**Table 1:** General genome features of XL1-Blue and Ajou 45.

Attribute	XL1-Blue	Ajou 45
Genome size (bp)	4,633,492	4,632,389
Number of contigs	1	1
G+C content (%)	50.8	50.8
CDS *	4443	4425
RNAs	110	110

* Coding DNA sequence.

**Table 2:** Functional and structural impacts of 11 selected mutations in well-characterized proteins in Ajou 45.

Gene Name	Encoded Protein	Function	Mutation Type	Amino Acid Changes	Predicted Functional Impact
*manB*	Phosphomannomutase	Converts mannose-6-phosphate to mannose-1-phosphate	995A > C	H332P	Possible change in enzyme activity affecting cell wall synthesis
*lacZ*	Beta-galactosidase	Hydrolyzes lactose into glucose and galactose	93 bp insertion; 906C > T	Frame shift	Altered expression or stability of lactose metabolism enzyme
*cynT*	Carbonic anhydrase	Catalyzes CO_2_ hydration	497C > T	G55D	Potential disruption in CO_2_ homeostasis
*prpB*	Methylisocitrate lyase	Converts methylisocitrate to succinate	332C > T	G111E	May affect the TCA cycle intermediate metabolism
*adhE*	Alcohol dehydrogenase	Converts acetaldehyde to ethanol	640C > T	A214A	Silent mutation
*carB*	Carbamate kinase	ATP-dependent carbamate phosphorylation	84C > T; 38C > T	E28E; S13N	May affect ATP binding or phosphorylation efficiency
*betB*	Betaine aldehyde dehydrogenase	Converts betaine aldehyde to glycine betaine	735C > T	S245S	Silent mutation
*argF*	Ornithine carbamoyl transferase	Catalyzes ornithine to citrulline conversion	131C > T	T44I	Potential impact on urea cycle and nitrogen metabolism
*ilvD*	Dihydroxy-acid dehydratase	Catalyzes steps in branched-chain amino acid synthesis	137C > T	M46I	Predicted impact on branched-chain amino acid production
*mtn*	Homocysteine S-methyltransferase	Methylates homocysteine to methionine	185C > T	G62E	May affect methionine biosynthesis
*cyaA*	Adenylate cyclase	Synthesizes cAMP from ATP	2655 bp deletion	Deletion	Possible reduction in cAMP levels, derepressing metabolic pathways

**Table 3:** Strains and plasmids used in this study.

Strain	Properties	Ref.
*E. coli* XL1-Blue	*endA*1 *gyrA*96(nal^R^) *thi*-1 *recA*1*relA*1 *lac glnV*44 F’[::Tn10 *proAB*^+^*lacI*^q^(△*lacZ*)M15] *hsdR*17(*rk*^-^*mk*^+^)	Stratagene
Ajou 45	β-carotene -overproducing mutant *E. coli* XL1-blue	This study
*E. coli* XL1-Blue_△*cyaA*	XL1-Blue, △*cyaA*	This study
**Plasmid**	**Properties**	**Ref.**
pUC19	Cloning vector. pMB1 origin. Inducible lac promoter, Ap^R^	NEB
pUC19-crtEXYIBZ	pUC19 expressing *crtEXYIBZ* from *P. agglomerans*	This study
pUCM	Cloning vector modified from pUC19; constitutive *lac* promoter, Ap^R^	This study
pMP11	pKD46 constitutively expressing Cas9, Ap^R^,	[18]
pgRNA	pBR322 constitutively expressing sgRNA, Cm^R^	[18]
pgRNA_cyaA	pBR322 constitutively expressing sgRNA targeting *cya*A gene, Cm^R^	This study

## Data Availability

Data are contained within the article.

## Supplementary Materials

The following supporting information can be downloaded at: https://www.mdpi.com/article/10.3390/ijms252312796/s1.
