# Supplementary material for: Genomic Insights into the Role of cAMP in Carotenoid Biosynthesis: Enhancing β-Carotene Production in Escherichia coli via cyaA Deletion
Source: Int J Mol Sci. 2024 Nov 28;25(23):12796. doi: 10.3390/ijms252312796 (PMC11641309; doi:10.3390/ijms252312796)
Supplement: Supplementary file 1 [file ijms-25-12796-s001.zip › ijms-3271122-supplementary.pdf]

Supplementary Material

Genomic Insights into the Role of cAMP in Carotenoid Biosynthesis: Enhancing β-Carotene Production in *Escherichia coli* via *cyaA* Deletion

Soon-Jae Kwon [1], Chan Bae Park [2,*] and Pyung Cheon Lee [1,*]

[1] Department of Molecular Science and Technology and Advanced College of Bio-Convergence Engineering, Ajou University, Woncheon-dong, Yeongtong-gu, Suwon 16499, Republic of Korea; soon87jae@ajou.ac.kr
[2] Department of Physiology, Ajou University School of Medicine, Suwon 16499, Republic of Korea
* Correspondence: chanbaepark@ajou.ac.kr (C.B.P.); pclee@ajou.ac.kr (P.C.L.); Tel.: +82-32-219-4560 (C.B.P.); +82-32-219-2461 (P.C.L.)

Supplementary Table S1. Comprehensive list of genetic variations in the genome in Ajou 45

Gene	Position	Changes of nucleotide
Translation elongation factor Tu	412,889	Insertion "T"
rRNA	455,978	Insertion "T"
Uptake hydrogenase small subunit precursor	738,034	Point mutation "C→T"
Exodeoxyribonuclease V gamma chain	921,864	Point mutation "A→G"
rRNA	1,154,954	Insertion "G"
Major fimbrial subunit StfA	1,421,462	Point mutation "T→G"
Mobile element protein	1,704,544	Insertion "C"
Non coding region	1,708,543	Insertion "C"
Non coding region	1,711,468 – 1,712,244	Deletion "777 bp"
Phosphomannomutase	1,758,150	Point mutation "A→C"
Mobile element protein	1,810,214 – 1,810,216	Deletion "3 bp"
	1,810,391	Point mutation "C→G"
	1,810,394	Point mutation "G→A"
Non coding region	1,810,398	Insertion "870 bp"
	1,810,399	Deletion "G"
	1,810,571 – 1,811,908	Deletion "1338 bp"
	1,925,226	Insertion "8 bp"
tRNA (5-methoxyuridine) 34 synthase	1,925,227	Deletion "T"
	1,925,238	Insertion "11 bp"
Mobile element protein	1,969,672	Insertion "G"
Mobile element protein	1,969,887	Deletion "A"
Non coding region	2,785,096	Deletion "G"
	2,785,276	Insertion "G"
Non coding region	3,491,928	Insertion "C"
Non coding region	3,496,228	Point mutation "T→C"
Mhp operon transcriptional activator	3,505,818	Point mutation "C→T"
Beta-galactosidase	3,507,356	Insertion "93 bp"
	3,508,230	Point mutation "C→T"
Carbonic anhydrase	3,514,575	Point mutation "C→T"
Methylisocitrate lyase	3,524,524	Point mutation "C→T"
Alcohol dehydrogenase	3,530,014	Point mutation "C→T"
Carbamate kinase	3,533,289	Point mutation "C→T"
	3,533,335	Point mutation "C→T"
FIG074102: hypothetical protein	3,534,881	Point mutation "C→T"
Non coding region	3,541,561 – 3,542,897	Deletion "1337 bp"
	3,542,995	Point mutation "C→T"

Betaine aldehyde dehydrogenase	3,546,875	Point mutation "C→T"
Putative inner membrane protein	3,557,086	Point mutation "G→A"
Mobile element protein	3,559,227	Insertion "2 bp"
	3,559,231	Insertion "C"
CFA/I fimbrial minor adhesin	3,568,850	Point mutation "C→T"
DUF1440 domain-containing membrane protein	3,571,332	Point mutation "C→T"
Periplasmic aromatic aldehyde oxidoreductase, iron-sulfur subunit YagT	3,572,687	Point mutation "A→G"
Hypothetical protein YagQ	3,576,432	Point mutation "C→T"
hypothetical protein	3,579,791	Insertion "1199 bp"
Ornithine carbamoyltransferase	3,584,698	Point mutation "C→T"
Dihydroxy-acid dehydratase	3,591,535	Point mutation "C→T"
Homocysteine S-methyltransferase	3,597,974	Point mutation "C→T"
rRNA	4,305,817	Deletion "C"
	4,306,814	Deletion "G"
Non coding region	4,523,491	Insertion "1629 bp"
Adenylate cyclase	4,523,492 – 4,526,146	Deletion "2655 bp"
rRNA	4,574,029	Deletion "G"
	4,574,486	Insertion "G"
	4,574,801	Deletion "C"

Supplementary Fig. S1.

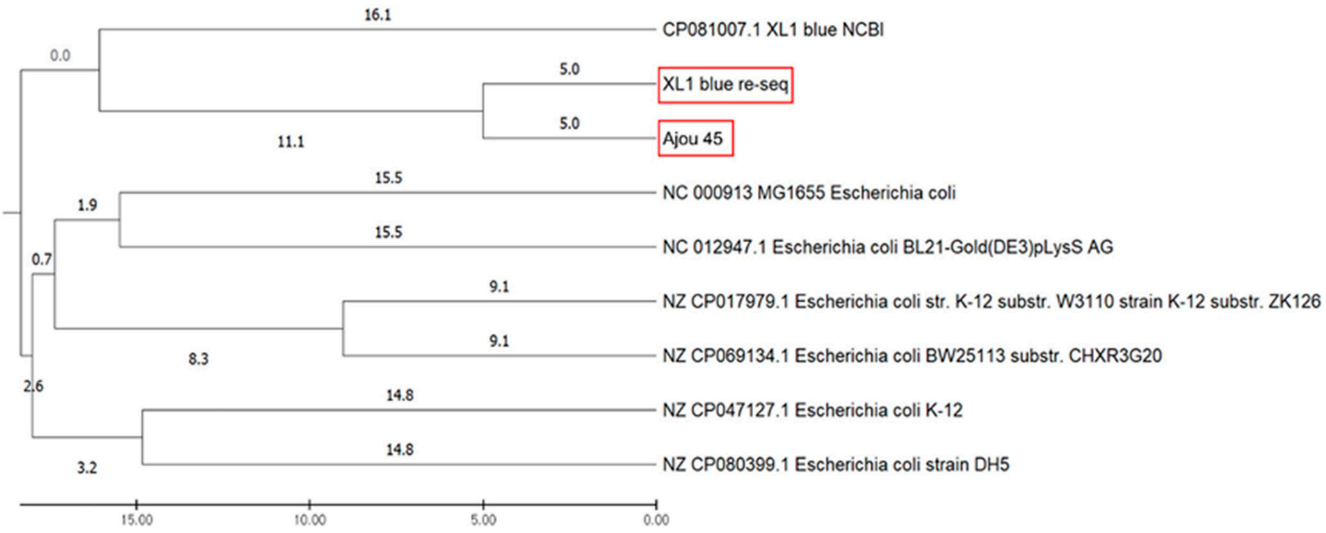

Figure S1. Phylogenetic analysis of β-carotene-overproducing mutant strain Ajou 45 and parental strain XL1-Blue. Phylogenetic analysis of β-carotene-overproducing mutant strain Ajou 45 and its parental strain XL1-Blue. The phylogenetic tree illustrates the evolutionary relationships among the resequenced genomes of Ajou 45 and XL1-Blue, compared with seven reference *Escherichia coli* genomes. The tree highlights that Ajou 45 and XL1-Blue form a distinct clade, signifying their close genetic relationship and confirming that Ajou 45 is derived from the parental XL1-Blue strain.

Supplementary Fig. S2.

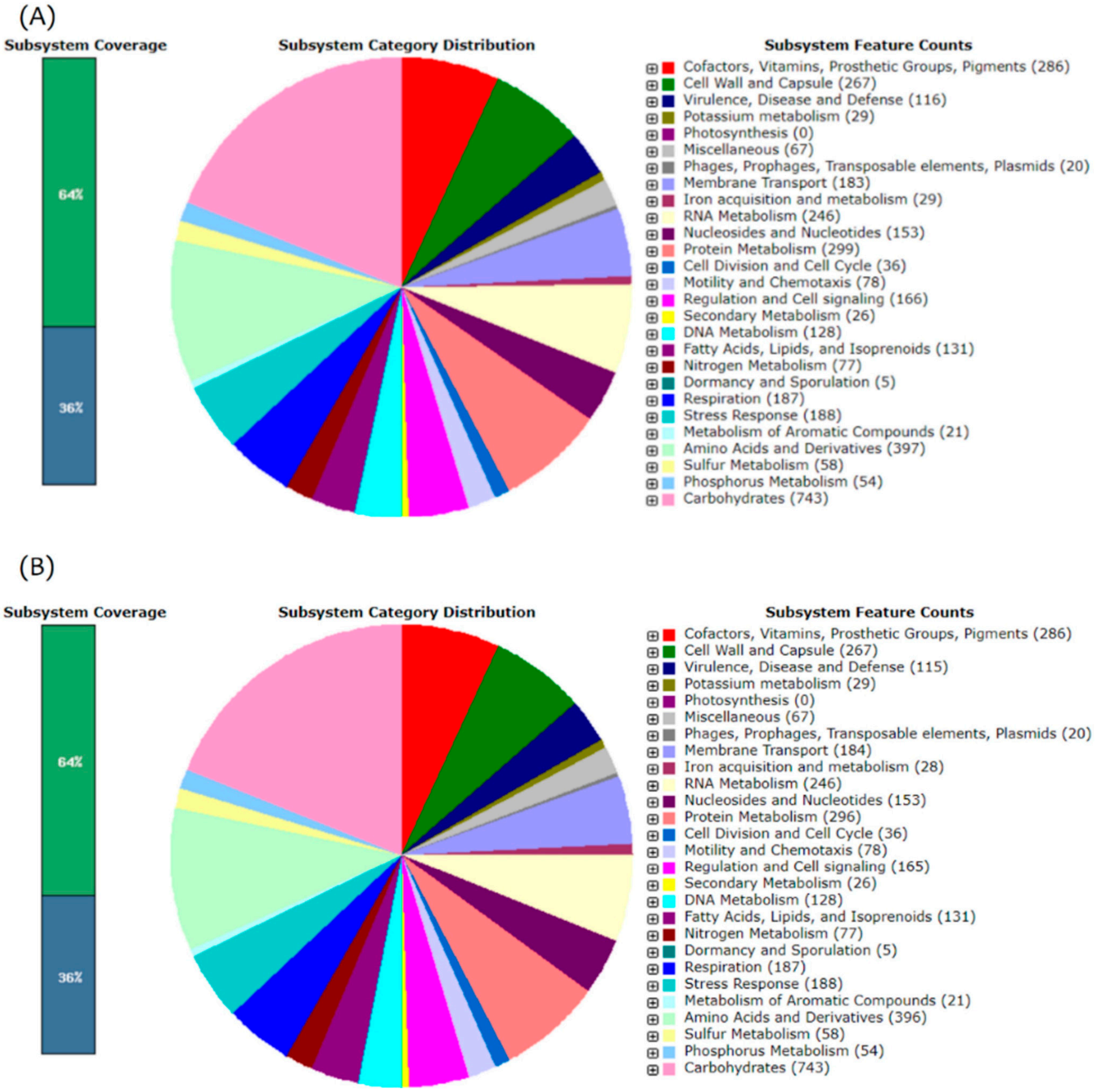

Figure S2. Subsystem category distribution and subsystem coverage for XL1-Blue and Ajou 45. (A) Subsystem category distribution and subsystem coverage for E. coli XL1-Blue based on annotated genome features. The pie chart displays the proportion of various subsystem categories, including carbohydrate metabolism, protein metabolism, RNA metabolism, and others. The bar on the left shows subsystem coverage, with 64% of features assigned to subsystems and 36% unassigned. (B) Subsystem category distribution and subsystem coverage for E. coli Ajou 45. The pie chart shows the proportion of various subsystem categories, similar to XL1-Blue. The bar on the left indicates that 64% of features are assigned to subsystems, and 36% remain unassigned.

Supplementary Fig. S3.

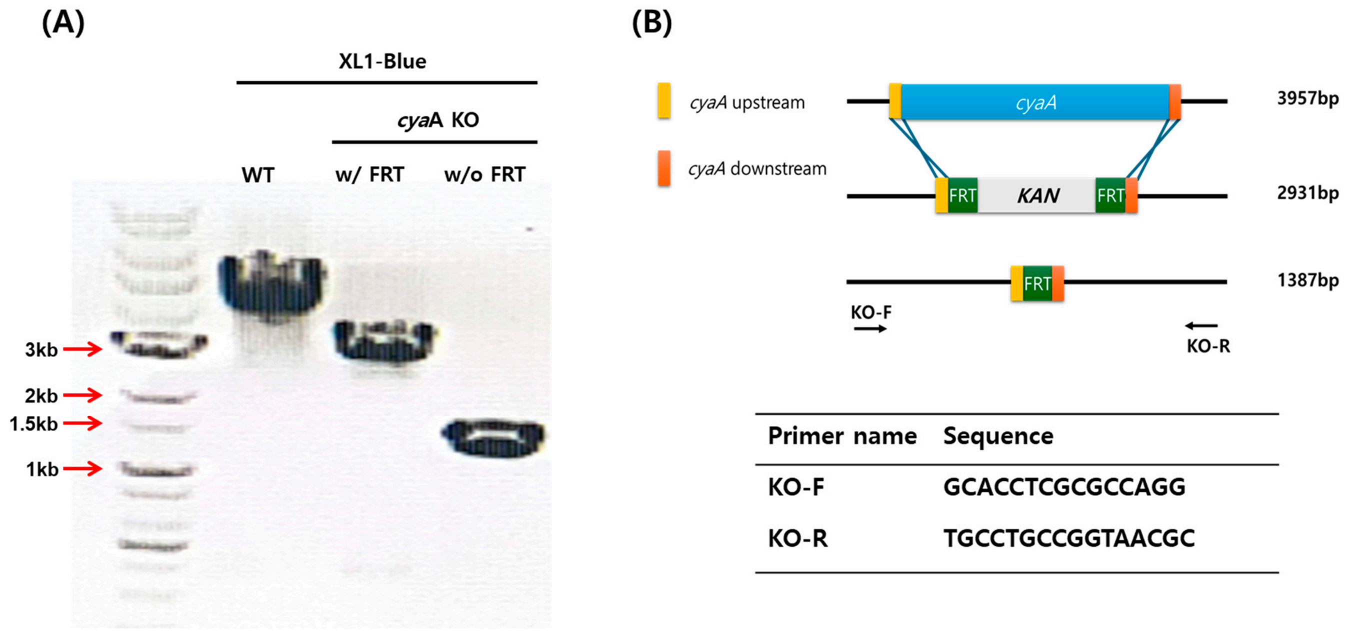

Primer name	Sequence
KO-F	GCACCTCGCGCCAGG
KO-R	TGCCTGCCGGTAACGC

Figure S3. Confirmation and schematic of cyaA deletion in XL1-Blue. (A) PCR analysis confirming cyaA deletion in XL1-Blue. Lane 1: molecular weight marker. Lanes 2: wild-type XL1-Blue showing intact *cyaA*. Lanes 3–4: XL1-Blue with smaller PCR products corresponding to *cyaA* deletion. Red arrows indicate DNA band sizes. (B) Schematic of the cyaA deletion strategy. The cyaA region was replaced with a kanamycin resistance gene flanked by FRT sequences, which were susequently excised, resulting in a single cyaA deletion in XL1-Blue.
